# Polysorbate 80 and *Helicobacter pylori*: a microbiological and ultrastructural study

**DOI:** 10.1186/1471-2180-12-217

**Published:** 2012-09-22

**Authors:** Natale Figura, Roberto Marcolongo, Giovanni Cavallo, Annalisa Santucci, Giulia Collodel, Adriano Spreafico, Elena Moretti

**Affiliations:** 1 Department of Internal Medicine, University of Siena, Siena, Italy; 2 Centre for Biochemical and Clinical Study of Rheumatic Diseases, Siena, Italy; 3 Qi s.r.l, Pomezia, Rome, Italy; 4 Biotechnology Department, University of Siena, Siena, Italy; 5 Department of Biomedical Sciences, Applied Biology Section, University of Siena, Siena, Italy; 6 Department of Clinical Medicine and Immunological Sciences, University of Siena, Siena, Italy

**Keywords:** Bacteria, Antibiotics, Chemoresistances, Polysorbate 80, Transmission electron microscopy

## Abstract

**Background:**

The frequent occurrence of chemoresistant strains reduces the chances of eradication of *H. pylori* infection and prompted the investigation of non-antibiotic substances active against this organism. Some surfactants enhance the effectiveness of antibiotics for their permeabilizing properties towards bacteria. We examined the antimicrobial activity to *H. pylori* of the surfactant polysorbate 80, used alone and in association with amoxicillin, clarithromycin, metronidazole, levofloxacin and tetracycline. We also aimed to study the ultrastructural alterations caused upon *H. pylori* by polysorbate 80, alone and in combination with antibiotics. Twenty-two *H. pylori* strains were tested using the broth dilution method. After incubation, broth from each dilution was subcultured onto agar enriched with foetal bovine serum to determine the minimum bactericidal concentration (MBC). Synergistic effect of polysorbate 80 with antibiotics was investigated by the broth dilution and disc diffusion techniques. Ultrastructural alterations of organisms treated with polysorbate 80, alone and in association with antibiotics were analyzed by transmission electron microscopy.

**Results:**

MBCs of polysorbate 80 ranged from 2.6 (1.1) μg/ml to 32 (0) μg/ml. Polysorbate 80 exerted a synergistic effect when associated with metronidazole and clarithromycin: polysorbate 80 and metronidazole MBCs decreased by ≥ 4 fold; clarithromycin MBCs for two resistant strains decreased by 20 and 1000 times. The principal alteration caused by polysorbate 80 consisted in the detachment of the outer membrane of bacteria.

**Conclusions:**

The bactericidal activity of polysorbate 80 and the synergistic effect of the association with metronidazole and clarithromycin could be useful in the treatment of *H. pylori* infection.

## Background

*Helicobacter pylori* is a microaerophilic gram-negative helical-shaped bacterium that infects approximately 30% of the population in developed countries and up to 90% of the population in developing countries [1,2].

The standard treatment of *H. pylori* infection, triple therapy, consists of two antibiotics and a proton pump inhibitor (PPI), or ranitidine bismuth citrate, administered for one or two weeks [3,4]. Amoxicillin, clarithromycin (or azithromycin), imidazoles (metronidazole or tinidazole), levofloxacin and tetracycline are the antibiotics used in the first and second line treatments. Options for third and subsequent line therapies include rifabutin and furazolidone-based regimes [5].

Recent protocols, such as the so-called sequential therapy, seem more successful than triple therapy; such treatment employs three antibiotics and a PPI and lasts for 10 days [6]. In 2011, Malfertheiner et al. [7] proposed a quadruple therapy (two antibiotics, tetracycline and metronidazole, PPI and bismuth) as a first line treatment because of the increasing prevalence of clarithromycin resistant strains.

Treatment failure is observed in 10%-23% of patients [4,8] and is mainly due to loss of antibiotic efficacy; in particular, the worldwide *H. pylori* antibiotic resistance rates in 2010 were 17.2% for clarithromycin, 26.7% for metronidazole, 11.2% for amoxicillin, 16.2% for levofloxacin, 5.9% for tetracycline and 9.6% for multiple antibiotics [9]. This dramatic fall in the eradication rates [10] strongly indicates the need to improve current therapeutic strategies and to develop new drugs, such as non-antibiotic substances [11-13]. Vitor and Vale [14] reviewed the study of alternative therapies, mainly probiotics and phytomedicine, for *H. pylori* infection. Probiotics attenuate the side effects of antibiotics and improve their efficacy; some plant extracts possess anti-*H. pylori* properties [14], but in this case, the active component should be identified, the mechanism of action and the potential toxicity for the patient explored, finally the possible resistance against these new phytotherapeutic agents addressed.

Among the numerous compounds with potential antibacterial properties, polysorbates, a class of substances derived from sorbitan, known with the commercial name of Tween®, are particularly appealing. In particular, polysorbate 80 is a nonionic surfactant used as an emulsifier in food, for example ice cream (where it is employed in concentrations of up to 0.5%). It is also used in bacterial broth cultures to prevent foam formation and as an excipient in numerous medications and vaccines against influenza to stabilize aqueous formulations. It is reputed to be a generally safe and well-tolerated compound.

These substances, in particular Tween 80, have been employed for their nature of surfactant to produce microemulsion systems with glycerol monolaurate as oil and organic acids as co-surfactant; such microemulsions caused a complete loss of viability of *Staphylococcus aureus* and *Escherichia coli*[15]*.* The potential antimicrobial activity of Tweens alone, however, was not explored. Other surfactants, such as dodecyl maltoside and octyl glucoside, enhanced the effectiveness of antibiotics used in the treatment of human pulmonary tuberculosis for their permeabilizing properties [16]. Finally, Huesca et al. [17] examined some substances, included Tween detergents, considered, in the past, efficacious treatments for peptic ulcer, and found that they were able to inhibit *H. pylori* receptor binding in vitro.

All these observations suggest that detergents could be useful in the treatment of *H. pylori* infection, although their potential antibacterial activity against *H. pylori* has not been examined yet. The aims of this study were: a) to determine the antimicrobial activity against *H. pylori* of polysorbate 80 and antibiotics most commonly used to eradicate *H. pylori* infection: amoxicillin, clarithromycin, metronidazole, levofloxacin and tetracycline; b) to find out whether the association of polysorbate 80 with antibiotics could increase their activity; c) finally, to investigate on the possible ultrastructural morphological alterations exerted upon *H. pylori* by polysorbate 80 (alone and in associations with clarithromycin and metronidazole), which could help explaining its mechanism of action.

## Results

### Characteristics of strains tested

The 22 strains tested include the different genotypes of *H. pylori* (*i.e. cagA-*positive or –negative) and different source of isolation, *i.e.* from patients with chronic gastritis only (CGO), duodenal ulcer (DU) and gastric carcinoma (GC). Fifteen strains were primary strains (that is isolated from patients naïve for eradication treatment), seven strains, isolated from patients unsuccessfully treated with the triple therapy, were named as secondary. The strain characteristics are reported in Table 1. Out of the 22 strains tested, six strains were isolated from patients with GC, three strains from cases of DU and the others from patients with CGO. Sixteen strains possessed the *cagA* gene; strain 328 Km was a *cagA-*negative isogenic mutant of the wild *cagA*-positive isolate 328 (Table 1).

**Table 1:** **Characteristics of *H. pylori *strains tested**

**Parameter**	***Helicobacter pylori*strains**																					
	**CCUG 17874**	**G50**	**G21**	**4Kb**	**DiSim**	**10 K**	**328**	328 Km*	**M/C-R1**	**M/C-R2**	**M/C-R3**	**Ap-R**	**3Cb**	**Marit**	**G27**	**17C7**	**Ba142**	**12A3**	**8C8**	**G104**	**Ver1**	**Ver2**
Presence of *cagA* gene	+	-	-	+	+	+	+	-	+	-	+	+	+	+	+	+	-	+	+	-	+	+
Pathology of patients	CGO	CGO	CGO	GC	DU	GC	CGO	CGO	CGO	CGO	CGO	DU	GC	CGO	DU	GC	CGO	GC	GC	CGO	CGO	CGO
Primary strain	Yes	Yes	Yes	Yes	Yes	Yes	Yes	Yes	No	No	No	No	Yes	No	Yes	Yes	Yes	Yes	Yes	Yes	No	No

* This is an isogenic *cagA* negative mutant of the wild strain 328.

CGO: chronic gastritis only; DU: duodenal ulcer; GC: gastric carcinoma.

### Determination of the chemosusceptibility of *H. pylori* strains to polysorbate 80 and antibiotics

The results of the chemosusceptibility tests are expressed in μg/mL and are reported in Table 2 as mean and standard deviation in parentheses. MBCs of polysorbate 80 ranged from 2.6 (1.1) to 32 (0) (Table 2); the MBC_50_ (the concentration at which ≥50% of strains were killed) was 16 (0). All strains were susceptible to amoxicillin (< 1.0 μg/ml) and MBCs ranged from 0.002 (0) to 0.6 (0.1); the MBC_50_ was 0.03 (0) (Table 2). Five secondary isolates (23.9%), were resistant to clarithromycin (> 1.0 μg/ml) (Table 2). Two strains presented a high level of resistance with MBC of 320 (0) and 2500 (0), while MBC of the other strains were 32 (0) for two strains and 64 (0) (Table 2). MBCs for the susceptible strains ranged from 0.01 (0) to 0.5 (0) (Table 2) and the MBC_50_ was 0.08 (0). Eight strains (36.3%, four strains were secondary) were resistant to metronidazole (>4 μg/ml) (Table 2); MBCs for resistant strains were 20.8 (7.2), 21.3 (9.2), 26.6 (9.2), 32 (0), 64 (0), 128 (0) for two strains and 170.6 (73.9) (Table 2). All strains, excepted one primary strain, were susceptible to levofloxacin (<2 μg/ml) (Table 2); MBCs ranged from 0.12 (0) to 0.5 (0) and the MBC_50_ was 0.25 (0) (Table 2). Finally, one primary and one secondary strains (9.0%) were resistant to tetracycline with MBC of 4 (0) and 6.6 (2.3); one strain was also resistant to metronidazole and clarithromycin, the other strain to metronidazole only. MBCs of tetracycline for the susceptible strains (< 4 μg/ml) ranged from 0.03 (0) to 2 (0) and the MBC_50_ was 0.25 (0).

**Table 2:** **MBCs of polysorbate 80, antibiotics and association of polysorbate 80 and antibiotics to the *H. pylori *strains tested; the values are expressed in μg/ml and reported as mean and standard deviation in parentheses**

**Drugs**	***Helicobacter pylori*strains**																					
	**CCUG 17874**	**G50**	**G21**	**4Kb**	**DiSim**	**10 K**	**328**	**328 Km**	**M/C-R1**	**M/C-R2**	**M/C-R3**	**Ap-R**	**3Cb**	**Mar-iot**	**G27**	**17 C7**	**Ba 142**	**12A3**	**8C8**	**G104**	**Ver1**	**Ver2**
Polysorbate 80	6.6 (2.3)	16 (0)	8 (0)	13.3 (4.6)	16 (0)	32 (0)	32 (0)	26.6 (9.2)	21.3 (9.2)	32 (0)	16 (0)	13.3 (4.6)	16 (0)	16 (0)	8 (0)	16 (0)	8 (0)	2.6 (1.1)	10.6 (4.6)	8 (0)	6.6 (2.3)	16 (0)
Amoxicillin	0.08 (0)	0.01 (0)	0.08 (0)	0.01 (0)	0.005 (0)	0.002 (0)	0.02 (0)	0.02 (0)	0.005 (0)	0.07 (.02)	0.01 (0)	0.005 (0)	0.01 (0)	0.07 (.02)	0.6 (.1)	0.1 (.04)	0.5 (0)	0.03 (0)	0.06 (0)	0.05 (.02)	0.04 (0)	0.08 (0)
Clarithromycin	0.25 (0)	0.01 (0)	0.01 (0)	0.08 (0)	0.08 (0)	0.11 (.05)	0.2 (0)	0.02 (0)	320 (0)	2500 (0)	0.03 (.01)	0.04 (0)	0.04 (0)	32 (0)	0.11 (.05)	0.06 (0)	0.5 (0)	0.06 (0)	0.05 (.02)	0.06 (0)	32 (0)	64 (0)
Metronidazole	32 (0)	0.4 (0)	2.6 (.3)	0.8 (0)	2.13 (0.9)	20.8 (7.2)	21.3 (9.2)	1.6 (0)	26.6 (9.2)	0.8 (0)	2.13 (.9)	0.8 (0)	0.67 (.23)	64 (0)	128 (0)	0.25 (0)	1.0 (0)	0.25 (0)	1.3 (.5)	0.25 (0)	128 (0)	170.6 (73.9)
Levofloxacin	0.32 (0)	0.27 (.09)	0.32 (0)	0.16 (0)	0.16 (0)	0.32 (0)	0.13 (.05)	0.16 (0)	0.25 (0)	0.32 (0)	0.16 (0)	0.32 (0)	0.13 (.05)	0.32 (0)	0.16 (0)	0.25 (0)	0.21 (.07)	0.12 (0)	0.5 (0)	2 (0)	0.25 (0)	0.21 (.07)
Tetracycline	2.0 (0)	0.25 (0)	1.67 (.58)	1.0 (0)	0.06 (0)	2.0 (0)	0.03 (0)	0.04 (.02)	0.06 (0)	0.06 (0)	0.25 (0)	0.25 (0)	0.05 (.02)	4 (0)	6.6 (2.3)	0.25 (0)	0.67 (.29)	0.5 (0)	0.5 (0)	2.0 (0)	0.32 (0)	0.16 (.13)
Polysorbate	4 (0)/0.08 (0)	6.6 (2.3)/0.01 (0)	3.1 (1.1)/0.08 (0)	4 (0)/0.01 (0)	4 (0)/0.005 (0)	3.1 (1.1)/0.002(0)	4 (0)/0.02 (0)	6.6 (2.3)/0.01 (0)	21.3 (9.2)/.01	16 (0)/0.02 (.01)	6.6 (2.3)/.01 (0)	4 (0)/0.01 (0)	4 (0)/0.01 (0)	4(0)/0.04 (0)	4(0)/0.02 (0)	3.1 (1.1)/0.04 (0)	3.1 (1.1)/0.3 (.14)	2.6 (1.1)/ 0.03 (0)	4 (0)/0.05 (.02)	4 (0)/0.04 (.01)	3.1 (1.1)/0.04 (0)	4 (0)/0.05 (.02)
80/Amoxicillin																						
Polysorbate 80/	2 (0)/0.016 (0)	4 (0)/0.02 (.01)	3.1 (1.1)/0.11 (.05)	4 (0)/0.01 (0)	8 (0)/0.05 (0)	4 (0)/0.01 (0)	8 (0)/0.025 (0)	8 (0)/0.05 (0)	4 (0)/20 (0)	8 (0)/2.5 (0)	3.1 (1.1)/0.005 (0)	4 (0)/0.02 (.01)	4 (0)/0.01 (0)	3.1 (1.1)/8.0 (0)	3.1 (1.1)/0.05 (0)	4 (0)/0.01 (0)	2 (0)/0.016 (0)	2.6(1.1)/0.02 (.01)	3.1 (1.1)/0.01 (0)	4 (0)/0.01 (0)	2.6(1.1)/3.1 (1.1)	4 (0)/8 (0)
Clarithromycin																						
Polysorbate 80/	2 (0)/2 (0)	4 (0)/0.25 (0)	4 (0)/1 (0)	8 (0)/0.2 (0)	4 (0)/0.8 (0)	4 (0)/8 (0)	4 (0)/0.25 (0)	32 (0)/0.8 (0)	8 (0)/4 (0)	8 (0)/0.1 (0)	4 (0)/1 (0)	8 (0)/0.2 (0)	16 (0)/0.67 (.23)	16 (0)/16 (0)	4 (0)/106.6 (37)	8 (0)/0.16 (.08)	8 (0)/0.2 (0)	2.6 (1.1)/0.08 (0)	6.6 (2.3)/0.8 (0)	8 (0)/0.16 (.08)	6.6 (2.3)/64 (0)	4 (0)/106.6 (37)
Metronidazole																						
Polysorbate 80/	8 (0)/0.16 (0)	16 (0)/0.32 (0)	6.6 (2.3)/0.32 (0)	10.6 (4.6)/1 (0.4)	13.3 (4.6)/0.13 (.46)	8 (0)/0.31 (0)	32 (0)/0.16 (0)	16 (0)/1.6 (0)	32 (0)/0.25 (0)	32 (0)/0.32 (0)	16 (0)/0.16 (0)	13.3 (4.6)/0.27 (.09)	9.33 (6.11)/0.13 (.05)	8 (0)/0.27 (.09)	8 (0)/0.16 (0)	16 (0)/0.25 (0)	8 (0)/0.21 (.07)	2.6 (1.1)/0.12 (0)	8 (0)/0.42 (.14)	8 (0)/2 (0)	6.6 (2.3)/0.25 (0)	16 (0)/0.16 (.13)
Levofloxacin																						
Polysorbate 80/	8 (0)/2 (0)	13.3 (4.6)/0.25 (0)	8 (0)/2 (0)	8 (0)/0.67 (.29)	16 (0)/0.08 (.03)	16 (0)/2 (0)	32 (0)/0.03 (0)	16 (0)/0.04 (.02)	32 (0)/0.06 (0)	32 (0)/0.03 (0)	13.3 (4.6)/0.25 (0)	8 (0)/0.16 (.08)	13.3 (4.6)/0.042 (.014)	16 (0)/4 (0)	6.6 (2.3)/6.6 (2.3)	8 (0)/0.16 (.08)	8 (0)/067 (.29)	2.6 (1.1)/0.42 (.14)	8 (0)/0.5 (0)	6.6 (2.3)/2 (0)	6.6 (2.3)/0.16 (0)	8 (0)/0.16 (.08)
Tetracycline																						

### Determination of the chemosusceptibility of *H. pylori* strains to polysorbate 80 used in association with clarithromycin or metronidazole

**Figure 1:**
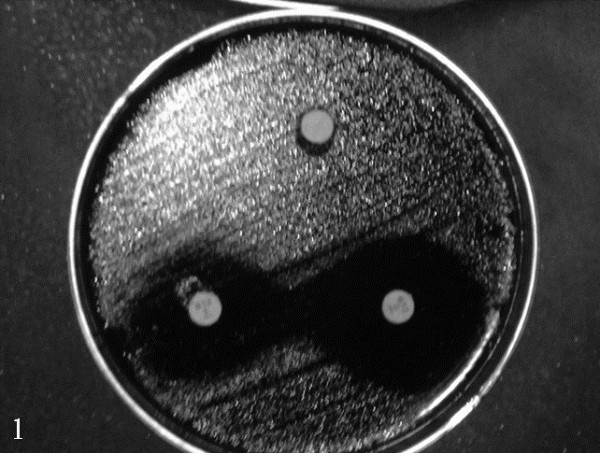
The combination of polysorbate 80 with metronidazole (disc on the right) increases the size of the growth inhibition halo; the disc on the left was charged with metronidazole alone and the disc at the top with polysorbate 80 alone.

The combination of polysorbate 80 with metronidazole increased the size of the growth inhibition halos (Figure 1); around the disk containing polysorbate 80, a minimal halo of complete inhibition of growth, ~1 mm, can be seen. Subculture tests showed the presence of another halo of about 4 mm contains developed dead bacteria. The same effect was observed when clarithromycin was assayed alone and with polysorbate 80 (data not shown). Halo sizes around discs charged with polysorbate 80 and amoxicillin, or levofloxacin, or tetracycline were not larger than those obtained with single antibiotics (data not shown). The synergistic effect of the association polysorbate 80/clarithromycin and polysorbate 80/metronidazole was confirmed by the broth dilution tests (Table 2). When used in association, the MBCs of polysorbate 80 decreased by 2–4 times and those of antibiotics by 2–16 times, compared to the respective MBCs of drugs used alone. The effect of the association of polysorbate 80 with amoxicillin, or levofloxacin, or tetracycline was negligible (Table 2).

### TEM analysis of CCUG 17874 and C/M-R2 *H. pylori* strains treated with polysorbate 80, alone and in association with clarithromycin and metronidazole

**Figure 2:**
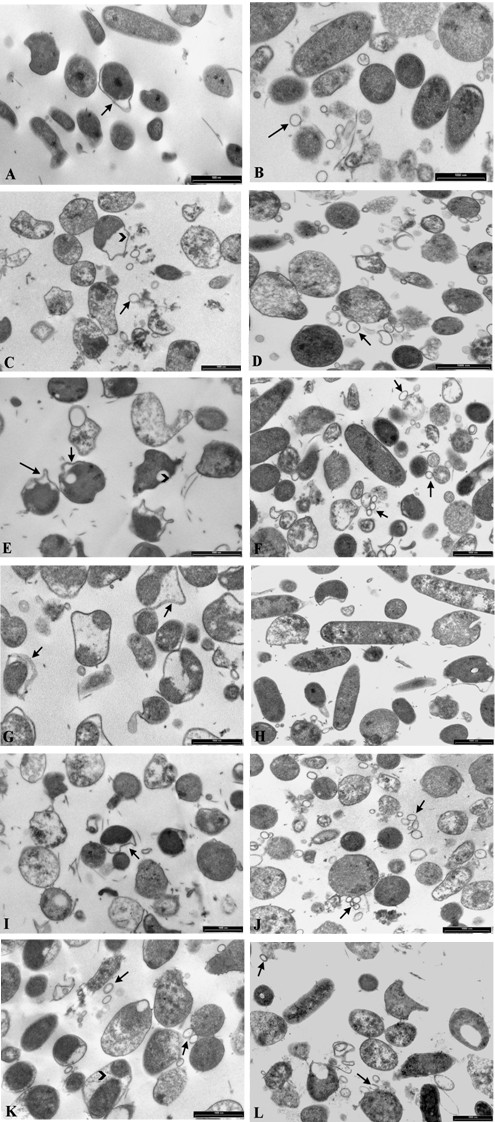
**TEM micrographs of CCUG 17874 (A, C, E, G, I, K) and M/C-R2 (B, D, F, H, J, L) ***H. pylori* strains. CCUG 17874 untreated bacteria (Figure 2A) show homogeneous cytoplasm and rare membrane/cytoplasm detachments (arrow). M/C-R2 untreated bacteria (Figure 2B) show homogeneous cytoplasm, flagella and vesicles (arrow). CCUG 17874 bacteria treated with polysorbate 80 (Figure 2C) are swollen and morphologically altered; cytoplasm is granular and detached from the inner membrane (arrow head); vesicles (arrow) are present. M/C-R2 bacteria treated with polysorbate 80 (Figure 2D) are swollen and morphologically altered; cytoplasm is not homogeneous and numerous vesicles are present (arrow). CCUG 17874 bacteria treated with clarithromycin (Figure 2E) show altered shape, typical “holes” in the cytoplasm (arrow head), membrane/cytoplasm detachment (arrows) and fragments of flagella. Some M/C-R2 organisms treated with clarithromycin (Figure 2F) have a conserved morphology, others show granular cytoplasm and altered membranes. Flagella and vesicles (arrows) are present. CCUG 17874 bacteria incubated with metronidazole (Figure 2G) are severely altered and show detachment of cytoplasm, often fragmented, from inner membrane (arrows). M/C-R2 bacteria treated with metronidazole (Figure 2H) are morphologically similar to control. CCUG 17874 treated with polysorbate 80 and clarithromycin (Figure 2I) displays alterations typical of organisms treated with the two substances used alone: swollen cells and detachment membrane/cytoplasm (arrow). M/C-R2 bacteria treated with polysorbate 80 and clarithromycin (Figure 2J) are mostly swollen, their cytoplasm is granular and numerous vesicles are present (arrows). CCUG 17874 strain treated with polysorbate 80 and metronidazole (Figure 2K) displays swollen bacteria, granular cytoplasm, presence of vesicles (arrows) and detachment of fragmented cytoplasm from the inner membrane (arrow head). M/C-R2 bacteria treated with polysorbate 80 and metronidazole (Figure 2L) are swollen; cytoplasm is granular and displays the presence of “holes”. Vesicles are present (arrows). Bars 2A-L: 1000 nm.

The ultrastructural characteristics of the two untreated strains appeared different from each other. CCUG 17874 *H. pylori* organisms showed homogeneous cytoplasm and rare detachment membrane/cytoplasm (Table 3, Figure 2A); ~ 5% of cells presented an altered profile. C/M-R2 organisms showed homogeneous cytoplasm and vesicles (Figure 2B). In both strains, flagella have been observed (Table 3).

**Table 3:** **Approximate percentages of organisms showing ultrastructural alterations observed in two *H. pylori *strains after treatment with polysorbate 80, clarithromycin, metronidazole, polysorbate 80/clarithromycin and polysorbate 80/metronidazole**

**Parameters**	**Controls**		**Polysorbate 80**		**Clarithromycin**		**Metronidazole**		**Polysorbate 80/**		**Polysorbate 80/**	
									**Clarithromycin**		**Metronidazole**	
	**CCUG 17874**	**C/M-R2**	**CCUG 17874**	**C/M-R2**	**CCUG 17874**	**C/M-R2**	**CCUG 17874**	**C/M-R2**	**CCUG 17874**	**C/M-R2**	**CCUG 17874**	**C/M-R2**
% Altered Shape	5	3	90	60	85	50	85	5	100	95	85	80
% Granular Cytoplasm	1	3	90	70	10	40	5	5	75	60	70	65
% Altered outer membrane	1	1	75	75	20	35	20	1	70	75	70	75
% Presence of “holes” in the cytoplasm	0	0	1	0	40	2	2	1	20	1	1	15
% Detachment membrane/cytoplasm	3	1	10	2	30	2	60	2	25	2	50	10
% Presence of flagella	YES	YES	NO	YES	YES	YES	NO	YES	YES	YES	NO	NO
% Presence of vesicles	-	+	++	++++	-	++	-	+	+/−	++++	++	++

To examine the ultrastructural characteristics of the organisms treated with the studied substances, the bacteria were incubated overnight with the single drugs and with antibiotics associated with polysorbate 80 at concentrations corresponding to the respective MBCs. In both strains treated with polysorbate 80 (Table 3), we observed swollen bacteria and alterations of the outer membrane (Figures 2C, 2D), particularly evident in CCUG 17874 *H. pylori* strain. The cytoplasm showed a typical granular texture; in both strains, we noted the presence of vesicles, which were more numerous in C/M-R2 strain.

The two strains challenged with clarithromycin showed different ultrastructural alterations. CCUG 17874 *H. pylori* strain (Figure 2E) was characterised by altered forms with typical “holes” in the cytoplasm and detachment of the inner membrane from the cytoplasm or cytoplasm retraction; flagella were observed, whereas vesicles were absent (Table 3). In C/M-R2 strain the morphology was conserved in about half of the analyzed bacteria (Figure 2F), whereas ~ 40% of cells showed granular cytoplasm and ~ 35% altered outer membrane. Flagella were observed and vesicles were present in C/M-R2 strain only (Table 3).

As far as the strains assayed with metronidazole are concerned, CCUG 17874 strain was characterised by organisms with severely altered shape and peculiar detachments between membrane and cytoplasm that often appeared fragmented (Figure 2G); flagella and vesicles were not observed in the sample (Table 3). C/M-R2 strain did not show any peculiar ultrastructural alterations after metronidazole treatment (Figure 2H).

In the samples treated with both polysorbate 80 and clarithromycin, the shape was altered in both bacterial strains and the synergic effect of the two compounds was evident (Figures 2I, 2J). The examination of CCUG 17874 strain revealed swollen cells, granular cytoplasm and altered outer membrane, typical alterations induced by polysorbate 80, together with detachment of the inner membrane from the cytoplasm and “holes” in the cytoplasm, typical effect of clarithromycin (Table 3). Flagella and rare vesicles were observed. C/M-R2 strain showed swollen bacteria with cytoplasm that gradually had lost its homogeneity; numerous vesicles and rare fragments of flagella were present (Table 3). The examination of CCUG 17874 strain treated with polysorbate 80 and metronidazole (Figure 2K) showed swollen bacteria with non-homogeneous cytoplasm, presence of vesicles (typical features of polysorbate 80 treatment) concomitant with peculiar detachments of the membrane from cytoplasm that often appeared fragmented (typical alterations caused by metronidazole). Vesicles were present, flagella were not observed (Table 3). C/M-R2 strain showed swollen bacteria with granular cytoplasm and the presence of vesicles (Figure 2L), all characteristics typical of polysorbate 80 treatment (Table 3); no flagella were found.

## Discussion

Chemoresistances are the main cause of therapeutic failure of *H. pylori* infection [18]. The occurrence of acquired resistances in such species is very high, because of certain characteristics that make *H. pylori* hypermutable [19]. Mutation rates in *H. pylori* are in fact 10–700 fold higher than that observed in other species, for instance *Escherichia coli*; in addition, the mechanisms of acquired chemoresistance in *H. pylori* include its significant genetic competence (*i.e.* the ability to recombine exogenous DNA) [19]. Stress conditions, such as the antibiotic treatment and the exposure to the gastric acid, induce numerous events in this species*,* which may end up enhancing the frequency of chemoresistances: a) the transcription and translation of natural competence genes, which increase the frequency of transformation; b) the transcription of a lysozyme-like protein, which promotes DNA donation from the neighbouring cells; c) the stimulation of DNA uptake machinery, which increases the import of foreign DNA [20]. An additional source of genetic exchange is the transfer of genomic islands by conjugative mechanisms [21]. If we consider that the antibiotics utilizable in the treatment of *H. pylori* infection are limited and that it is mandatory to use them in combination of two or three at a time to be efficacious, the obvious conclusion is that in a few years physicians might lack effective antibiotics.

These observations prompted various researchers to investigate non-antibiotic compounds for their antimicrobial activity against *H. pylori.* Phytomedicine holds great promise for the treatment of *H. pylori* infection; however, it did not overcome the problem of resistance to the current antibiotics, nor has potentiated the antibiotic treatment [22]. The results of the present study showed that polysorbate 80 is bactericidal towards *H. pylori* with MBCs that could easily be achieved in the stomach. In addition, experiments in animals have established that polysorbate 80’s toxic dosages are very high: the equivalent toxic dosage for human beings is > 350 g a day for three days [23]. The best demonstration that such substance is safe and well tolerated comes from the observation that it became part of most foods in Europe and America, where each person ingests about 100 mg of polysorbate 80 in foods per day [24].

As polysorbate 80 is a detergent, it is likely that it exerts an antimicrobial activity against *H. pylori* by reacting with the bacterial outer membrane. Thus, in order to shed light upon its mechanism of action, we examined by TEM strains exposed to polysorbate 80, alone and associated with metronidazole and clarithromycin, the two antibiotics with which it showed a synergistic effect.

The observed morphological alterations in all samples treated with polysorbate 80 are conceivably caused by the detergent properties of this compound. Every time the bacteria have been treated with polysorbate 80, typical and recurrent ultrastructural anomalies have been detected, namely alterations of the bacterial shape, swelling of the organisms, loss of the normal and homogeneous cytoplasmic structures, anomalies in the bacterial envelope especially in the outer membrane and the presence of numerous vesicles. In the CCUG 17874 strain the vesicles were detectable only after polysorbate 80 treatments, used alone and in combination with antibiotics. Different is the situation for the M/C-R2 strain, in which the vesicles were present in the control (untreated) samples, but they became more numerous in the treated specimens. The ability of some *H. pylori* strains to naturally produce vesicles is well known [25]; however, after treatment of this strain with polysorbate 80, the number of vesicles definitely increased. The vesicles most likely originate from the outer membrane of bacteria: in the presence of detergents, the phospholipid bilayer is disrupted and micellae-like structures are produced. It is noteworthy that in both strains treated with polysorbate 80 we observed similar ultrastructural alterations, such as swelling of the organisms, alterations of the outer membrane and cytoplasm and presence of vesicles. A different behaviour of both strains was detected after treatment with antibiotics. Clarithromycin induced peculiar ultrastructural alterations in CCUG 17874 strain, namely typical “holes” in the cytoplasm, whereas in C/M-R2 strain we observed organisms with granular cytoplasm and altered envelopes. Similar modifications were described in strains treated with a different macrolide, erythromycin [26]. Metronidazole caused severe alterations in CCUG 17874 strain whereas it did not alter the normal morphology in the C/M-R2 strain, as also observed by Armstrong et al. [26].

In the specimens treated with antibiotics in association with polysorbate 80, the bacteria showed a combination of ultrastructural anomalies typical of the organisms challenged separately with the antibiotics, but at concentrations reduced by approximately four-times*.*

The observation of a synergistic effect of polysorbate 80 associated with metronidazole and clarithromycin deserves some comments. We have observed a reduction of metronidazole’s MBCs when the drug was associated with polysorbate 80, independently of whether strains were metronidazole susceptible or resistant. It is likely that the mechanism of synergy consists in an increased influx or improved bioavailability of such chemotherapic, determined by the damage of the outer membrane exerted by polysorbate 80 (as shown by TEM). This interpretation is supported by the observation that resistance to metronidazole might be overcome with increased doses of drug [27].

Out of the eight metronidazole resistant strains used to evaluate the outcome of associations, in three cases, polysorbate tested with metronidazole reduced the MBCs of the chemoterapic to concentrations at which strains can be considered susceptible, *i.e.* ≤ 4 μg/mL. The main mechanism of metronidazole resistance in *H. pylori* consists in mutations in *rdxA* and *frxA* genes, which encode an NADPH nitroreductase and an oxidoreductase, respectively [28]; the drug has to be reduced by bacterial reductive enzymes to exert its antimicrobial activity. Some researchers, however, claim that the first step to the development of metronidazole resistance consists in the overexpression of *hefA* gene, which encodes for an efflux pump [29]. Efflux pumps are very common amongst bacteria, including *H. pylori*, and protect them from the possible toxic effects of metabolite or antibiotic accumulation [30,31]. One component of a family of multidrug efflux transporters [32], widespread only among Gram-negative bacteria, is localised in the outer membranes [33]. Since it has been shown that the inactivation of any constituent of the efflux mechanism can abrogate the function of the entire group of efflux systems [29], we have speculated that the damage of the outer membrane exerted by polysorbate 80 could have caused the loss of such efflux transporter in our strains, thus impairing the mechanism of resistance. The strains still resistant to metronidazole even after treatment with polysorbate 80 could also have undergone a mutation of the reduction systems, *i.e.* it had a double mechanism of resistance.

The increased susceptibility to clarithromycin used in combination with polysorbate 80 could also be due to an augmented permeability of membranes exerted by the detergent. The main constituent of the outer membrane in Gram-negative bacteria is lipopolysaccharide (LPS); it coats the cell surface and works to exclude large hydrophobic compounds, such as antibiotics, from invading the cell. LPS has a significant role in membrane transport: the lipid compositions of LPS and the associated proteins have a strong impact on the sensitivity of bacteria to many types of antibiotics [34]. Unlike small hydrophilic antibiotics, large lipophilic agents, such as macrolides, have difficulty in diffusing through the LPS. Previous studies indicate that membrane permeabilizers, such as Tris/EDTA, polymyxin B *etc.*, have the ability to increase the levels of antibiotic inflow [34] and consequently the sensitivity of Gram-negative bacteria to hydrophobic antibiotics, including macrolides [35,36]. In this study, two strains were highly resistant to clarithromycin, with MBCs of 320 μg/mL and 2500 μg/mL. In the presence of polysorbate 80, clarithromycin’s MBCs decreased by 16 times and 1000 times, respectively, *i.e.* to 20 μg/mL and 2.5 μg/mL, which still are in the range of resistant values (threshold = 1 μg/mL). In these cases, we hypothesize the concomitance of two mechanisms of resistance. In a large number of bacterial species, in fact, the existence of drug-resistant strains is due to modifications in the lipid or protein composition of the outer membrane, which work in synergy with other resistance mechanisms [34]. Point mutations in 23S rRNA normally account for the development of resistance to clarithromycin in *H. pylori* and reduce the chances of eradication when the classical triple therapy is employed [37]. It is likely that in our strains the presence of an efflux apparatus cooperates with putative 23S rRNA mutations to make these two strains highly resistant to clarithromycin [38]. Polysorbate 80 conceivably increased their sensitivity by destroying the outer membrane; strains, however, were still resistant because of the existence of another putative mechanism, such as ribosome mutation.

A plausible explanation for the observation that the association of polysorbate 80 with amoxicillin, levofloxacin and tetracycline was not synergistic may consist in the sizes and hydrophilic nature of antimicrobials. Macromolecules such as clarithromycin, which hardly penetrate into bacteria using the lipid layer, exploit the alterations of the bacterial outer membranes to diffuse into microorganisms. Small hydrophilic antibiotics, such as β-lactams, tetracycline, fluoroquinolones *etc.*, use porin channels to cross the outer membrane and diffuse very well [39]. For this reason, they do not take advantage by the disruption of membranes; thus their association with polysorbate 80 is indifferent.

## Conclusions

In conclusion, polysorbate 80 shows a bactericidal activity against *H. pylori* and exerts a synergistic effect with some chemotherapics. We therefore propose such compound for the treatment of *H. pylori* infection in association with antibiotics.

## Methods

### Determination of MBC

The 22 strains used are listed in Table 1. The whole study was conducted following the approval of the local University Hospital Ethics Committee. All patients gave a written informed consent prior to inclusion of strains isolated from them in the study. Bacterial suspensions were stored in glycerol broth at −80°C until the MBC determination was carried out. Suspensions were thawed and subcultured twice in selective *Brucella* agar plates (Pylori plates, BioMérieux, Italia S.p.A., Rome, Italy.) containing 10% foetal calf serum and 10 mg/L of each vancomycin, trimethoprim, and amphotericin B and 5 mg/L of cefsulodin. Plates were incubated in jars with a microaerobic environment generated using Campy Pak sleeves (Oxoid Ltd., Basingstoke, England).

Polysorbate 80 and antibiotics -amoxicillin, clarithromycin, metronidazole, tetracycline and levofloxacin- (Sigma Aldrich-Milan, Italy) were dissolved in sterile water containing (when necessary) 4% of DMSO, sterilized by filtration and double diluted in *Brucella* broth containing 10% foetal calf serum, 10 mg/L of each vancomycin, trimethoprim, and amphotericin B and 5 mg/L of cefsulodin (to avoid contaminations). One microwell contained plain broth and was the control. Tests were carried out in triplicate in a final volume of 0.1 mL, using Microtiter® plates. *H. pylori* suspensions were prepared starting from cultures on *Brucella* agar with 10% foetal calf serum incubated in a microaerobic environment for 48 h. The bacterial suspensions were then added to each microwell at a final concentration of approximately 10^6^ colony-forming units per mL. After 24 h of incubation under microaerobic conditions at 37°C, 3 μL of broth from each dilution were deposited onto Brucella agar plates, which were incubated for 3–5 days in a microaerobic atmosphere at 37°C. The lowest concentration in broth, for which the subculture on agar showed complete absence of growth, was considered the MBC. Results are the average of three determinations.

### Determination of antimicrobial activity of polysorbate 80 associated with antibiotics

Tests to evaluate the possible synergistic effect of polysorbate 80 associated with antibiotics were performed on all strains. Two methods have been used, the disc diffusion and the broth dilution techniques. Briefly, blood-agar plates were seeded using a swab with a suspension of the type strain CCUG 17874 or the strain C/M-R2, whose density corresponded to McFarland no. 4 opacity standard. After the surface was dried, three paper discs were deposited on each plate, one disc was charged with the antibiotic (amoxicillin 2 μg, clarithromycin 15 μg, metronidazole and levofloxacin 5 μg each and tetracycline 10 μg), one with polysorbate 80 (0.4 mg) and the third one with both drugs, polysorbate 80 and antibiotic, at the same concentration present in the discs charged with single antibiotics. After a 3-day incubation in microaerobic environment at 37°C, plates were inspected and the halos of growth inhibition measured. The broth dilution test was carried out as follows: after the first drug was diluted, the second drug was added to each well of the first row containing different concentrations of the first compound; afterwards, the dilution of the second compound was carried out. Concurrently, we determined the MBC of the single substances. Tests were performed in triplicate.

### Ultrastructural analysis of *H pylori* with transmission electron microscopy (TEM)

For the ultrastructural analysis two strains of *H. pylori* were used: CCUG 17874 (metronidazole resistant type strain, isolated from a chronic gastritis case) and C/M-R2 (clarithromycin resistant clinical strain isolated from a chronic gastritis case). These two strains were treated with: 1-polysorbate 80, 2-clarithromycin, 3- metronidazole, 4- polysorbate 80 and clarithromycin, 5- polysorbate 80 and metronidazole. The other antibiotics were not tested because they did not exert any synergistic effect when examined in association with polysorbate 80.

The bacterial suspensions, after overnight incubation with the drugs at the concentrations corresponding to the respective MBCs and MBCs of their associations, were washed in phosphate-buffered saline (PBS), fixed in cold Karnovsky fixative and maintained at 4°C for 2 h. Fixed organisms were washed in 0.1 mol/L cacodylate buffer (pH 7.2) for 12 h at 4°C and postfixed in 1% buffered osmium tetroxide at 4°C for 1 h. Then the samples were washed in 0.1 mol/L cacodylate buffer (pH 7.2) for at least 2 h at 4°C, dehydrated in a series of ethanol (50%, 75%, 95%, 100%), exchanged through propylene oxide and embedded in Epon Araldite. Ultra-thin sections were obtained with a Supernova ultramicrotome (Reickert Jung, Vienna, Austria) with diamond knife, mounted on copper grids, stained with uranyl acetate and lead citrate and observed and photographed with a Philips EM208 TEM (Philips Scientifics, Eindhoven, The Netherlands).

A minimum of 500 bacteria per sample were analyzed and the anomalies related to the bacterial morphology (altered shape), the cytoplasm texture (granular cytoplasm, “holes” in the cytoplasm), the envelopes (altered envelopes, outer membrane detachment from the cell wall and cytoplasm detachment from the inner membrane), the presence of flagella and vesicles were quantified. The experiments were performed twice.

## Competing interests

The authors declare that they have no competing interests.

This work was supported in part by Over Italia, S.r.l., Sora (Frosinone) (Contract of research between Over and University of Siena N. 52514/III-17) Italy. Over s.r.l. is the owner of the patent PCT/IT2011/000175.

## Authors’ contribution

NF: substantial contributions to conception and design, bacterial culture, susceptibility tests and manuscript writing. EM: substantial contributions to conception and design electron microscopy and manuscript writing. RM and GC substantial contributions to conception and design. GC: electron microscopy, revision of the manuscript. AS and AS: contribution of interpretation of the data. All the authors revised the manuscript and gave their final approval.
